# Supplementary material for: Association between oral microbiome and seven types of cancers in East Asian population: a two-sample Mendelian randomization analysis
Source: Front Mol Biosci. 2023 Nov 21;10:1327893. doi: 10.3389/fmolb.2023.1327893 (PMC10702768; doi:10.3389/fmolb.2023.1327893)
Supplement: Supplementary file 1 [file DataSheet1.ZIP › Supplementary Materials/MR plots of saliva/saliva═╝/Pancreatic cancer/pheno.1421_to_pancreatic cancer_leave_one_out.pdf]

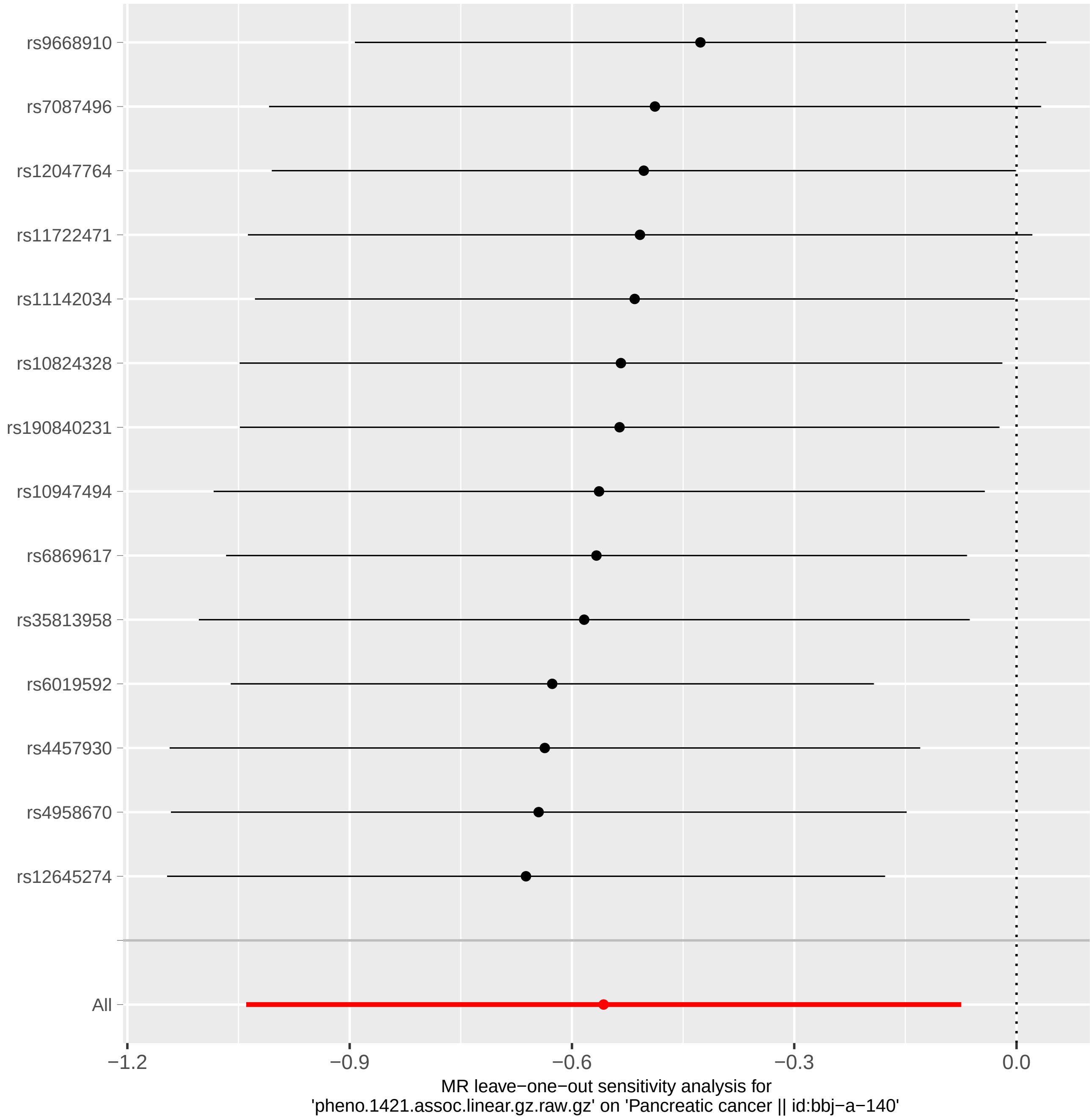
rs9668910
rs7087496
rs12047764
rs11722471
rs11142034
rs10824328
rs190840231
rs10947494
rs6869617
rs35813958
rs6019592
rs4457930
rs4958670
rs12645274
All
−1.2
−0.9
−0.6
−0.3
0.0
MR leave−one−out sensitivity analysis for
'pheno.1421.assoc.linear.gz.raw.gz' on 'Pancreatic cancer || id:bbj−a−140'
